# Contrasting dynamics of leaf potential and gas exchange during progressive drought cycles and recovery in *Amorpha fruticosa* and *Robinia pseudoacacia*

**DOI:** 10.1038/s41598-017-04760-z

**Published:** 2017-06-30

**Authors:** Weiming Yan, Shuxia Zheng, Yangquanwei Zhong, Zhouping Shangguan

**Affiliations:** 1 State Key Laboratory of Soil Erosion and Dryland Farming on the Loess Plateau, Northwest A&F University, Yangling, Shaanxi 712100 P.R. China; 2 State Key Laboratory of Vegetation and Environmental Change, Institute of Botany, Chinese Academy of Sciences, Beijing, 100093 China

## Abstract

Leaf gas exchange is closely associated with water relations; however, less attention has been given to this relationship over successive drought events. Dynamic changes in gas exchange and water potential in the seedlings of two woody species, *Amorpha fruticosa* and *Robinia pseudoacacia*, were monitored during recurrent drought. The pre-dawn leaf water potential declined in parallel with gas exchange in both species, and sharp declines in gas exchange occurred with decreasing water potential. A significant correlation between pre-dawn water potential and gas exchange was observed in both species and showed a right shift in *R. pseudoacacia* in the second drought. The results suggested that stomatal closure in early drought was mediated mainly by elevated foliar abscisic acid (ABA) in *R. pseudoacacia*, while a shift from ABA-regulated to leaf-water-potential-driven stomatal closure was observed in *A. fruticosa*. After re-watering, the pre-dawn water potential recovered quickly, whereas stomatal conductance did not fully recover from drought in *R. pseudoacacia*, which affected the ability to tightly control transpiration post-drought. The dynamics of recovery from drought suggest that stomatal behavior post-drought may be restricted mainly by hydraulic factors, but non-hydraulic factors may also be involved in *R. pseudoacacia*.

## Introduction

Water availability is one of the principal factors limiting terrestrial biological activity in ecosystems^1^. Drought, caused by declining soil water availability, has become more severe and frequent in several areas in the world due to climate change^2–4^. Periods of severe drought that have caused major declines in net primary productivity across the world, driving large-scale forest mortality events, have received much attention in recent years^5, 6^. Leaf relations may be a primary factor controlling gas exchange under drought conditions^7–11^. Thus, it is important to understand the regulation mechanisms of leaf water potential (Ψ_leaf_) on gas exchange during recovery from drought and over recurrent drought cycles.

Ψ_leaf_ is an important, sensitive measure for evaluating water availability and shows substantial reductions under prolonged drought stress^12–14^. Maintaining Ψ_leaf_ within an operational range is essential to plant metabolism and survival, and the response of Ψ_leaf_ to soil water depletion plays a crucial role in overcoming drought conditions^9^. The stomata act as pressure regulators of Ψ_leaf_, controlling the flow rate to avoid sharp decreases in the Ψ_leaf_^15^. Rapid stomatal closure is associated with an increased Ψ_leaf_ but may sometimes occur too late to prevent the early loss of Ψ_leaf_ and the associated loss of leaf hydration, which trigger further closing of the stomata^16^. Therefore, a significant correlation between Ψ_leaf_ and stomatal conductance (g_s_) is often observed under drought stress^16–18^, but the correlations between Ψ_leaf_ and both photosynthesis rate (*A*) and transpiration rate (*E*) require further study^19^. Understanding the correlations of *A* and g_s_ with Ψ_leaf_ would permit an estimation of the limitation of photosynthesis in plants grown under drought stress via stomatal closure and reduced mesophyll conductance or metabolic impairment^17, 20^. However, it remains unclear how Ψ_leaf_ controls gas exchange during the re-watered period and subsequent drought and whether the relations between Ψ_leaf_ and gas exchange are shifted during recurrent drought cycles^11, 21^.

When plants suffer drought, plant growth is often the first process to be affected due to the acute sensitivity of cell turgor and the effects of cell division, enlargement and differentiation^22, 23^. Stomatal closure, which decreases water loss and photosynthesis, delays the decline of water potential^24^ and is involved in the regulation of hormone signaling and water potential^25^. The decline of *A* in response to mild-to-moderate water stress occurs because of increased diffusive resistance within the leaf as well as decreased mesophyll conductance of CO_2_^26^. However, under severe stress, both diffusion and biochemical limitations limit *A* ^11, 26^, and it is generally accepted that there is a shift from limitation due mostly to decreased CO_2_ diffusion with mild-to-moderate water stress to a combination of diffusion and biochemical limitation under severe water stress^27, 28^.

Although the response of leaf gas exchange to drought has received much attention, less attention has been given to the underlying mechanisms of plant recovery from severe drought^26^. Water potential is one of the primary governors of leaf gas exchange under drought conditions, and the recovery of leaf gas exchange in plants shows a strong correlation with Ψ_leaf_ recovery, although the timing and mechanism of recovery are not clear^29^. Thus, an accurate description of the relationship between gas exchange performance and water potential during recovery from drought and a subsequent drought cycle would significantly improve our understanding of plant responses to drought stress.

In this study, we monitored dynamic changes in gas exchange and water potential simultaneously in two woody species, *Amorpha fruticosa* L. and *Robinia pseudoacacia* L. These two species are preferred species for afforestation on the Loess Plateau due to their strong drought resistance, and have been planted widely in this region. The large-scale afforestation of *R. pseudoacacia* has caused significant problems, such as a dry soil layer and trees that are small at maturity, due to higher water consumption; these problems are not observed following *A. fruticosa* afforestation. Thus, the primary goals of this study were to compare the different physiological responses of *R. pseudoacacia* and *A. fruticosa* under prolonged drought, to measure the association between the Ψ_leaf_ and gas exchange during the drought cycle and to determine whether the relationship between these parameters shifts in a second drought cycle. Furthermore, studies on the recovery capabilities of the two species after drought are needed. Therefore, a secondary goal was to assess and compare the mechanisms of the recovery of physiological functions in these two plant species during recovery from severe drought after re-watering. This study reveals the relationship between Ψ_leaf_ and gas exchange in recurrent drought cycles and the factors limiting the recovery of gas exchange after drought.

## Results

### The physiological response of plants to recurrent drought

**Figure 1:**
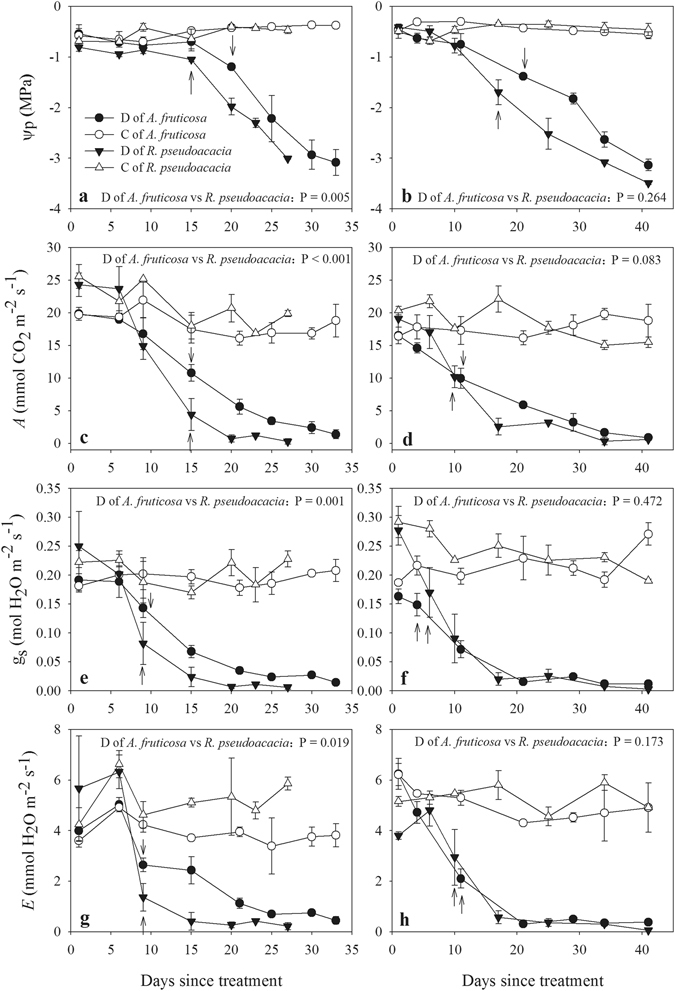
Comparison of pre-dawn leaf water potential (Ψ_p_), net photosynthesis (*A*), stomatal conductance (g_s_) and leaf transpiration (*E*) during drought cycles 1 (**a**,**c**,**e**,**g**) and 2 (**b**,**d**,**f**,**h**) between *A. fruticosa* and *R. pseudoacacia*. Panels c and d in graphic symbols represent plants in the control and drought groups, respectively; the arrow indicates the threshold of statistical significance at P < 0.05 between the drought and control plants of both species, and the error bars represent standard error, n = 3.

The loss of soil water under progressive drought in both species was followed by a decline of pre-dawn leaf water potential (Ψ_p_) and gas exchange (Fig. 1). Ψ_p_ showed no significant decrease up to 15 days after treatment, whereas compared with Ψ_p_, gas exchange parameters showed a rapid decrease in both species. Ψ_p_ and gas exchange parameters exhibited no significant difference in the control plants during the experiment.

**Figure 2:**
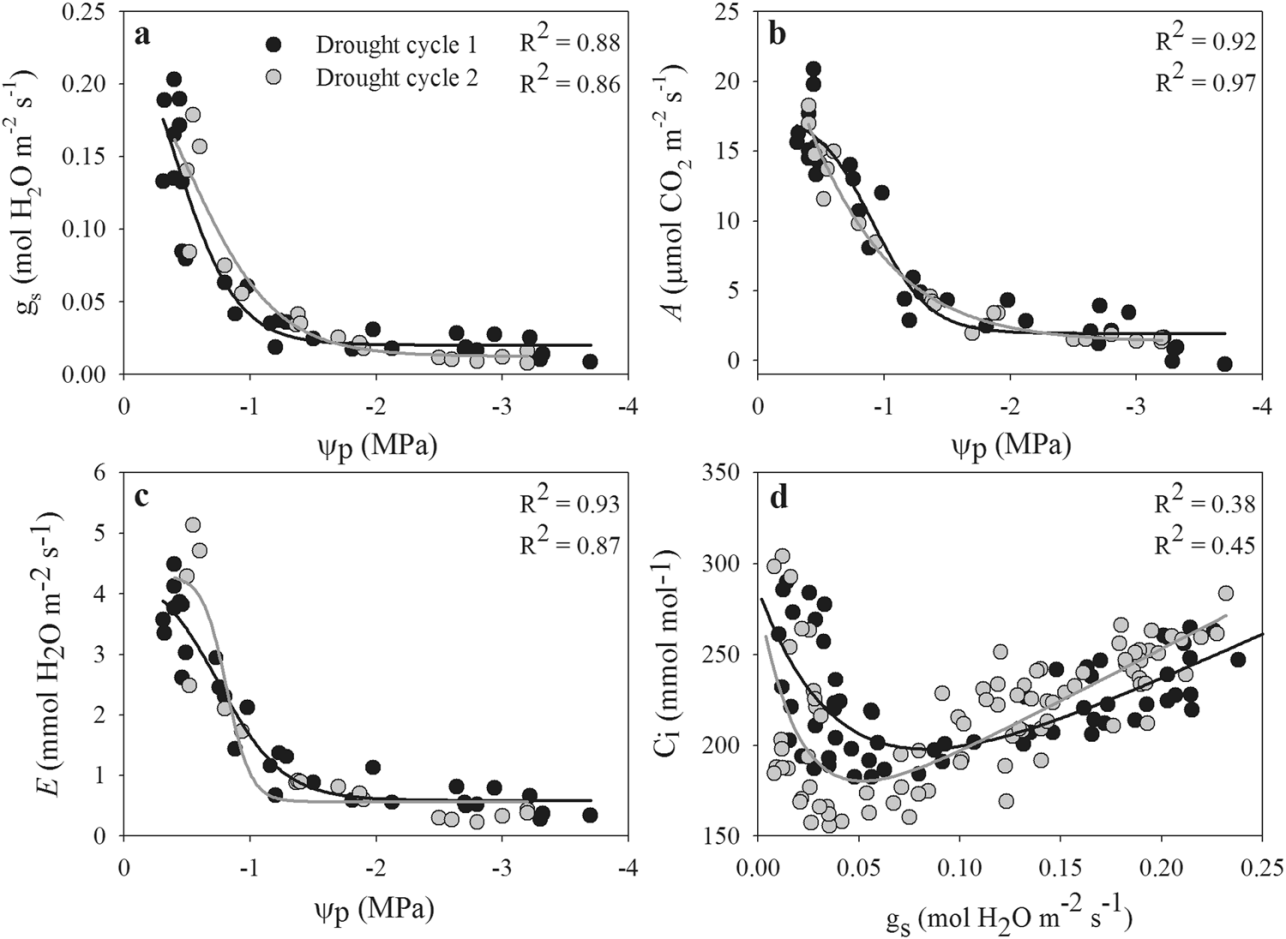
Relationships of measured g_s_, *A* and *E* with Ψ_p_ and of intracellular CO_2_ concentration (Ci) with g_s_ in *A. fruticosa* of drought plants during drought cycles 1 (black circles) and 2 (grey circles).

**Figure 3:**
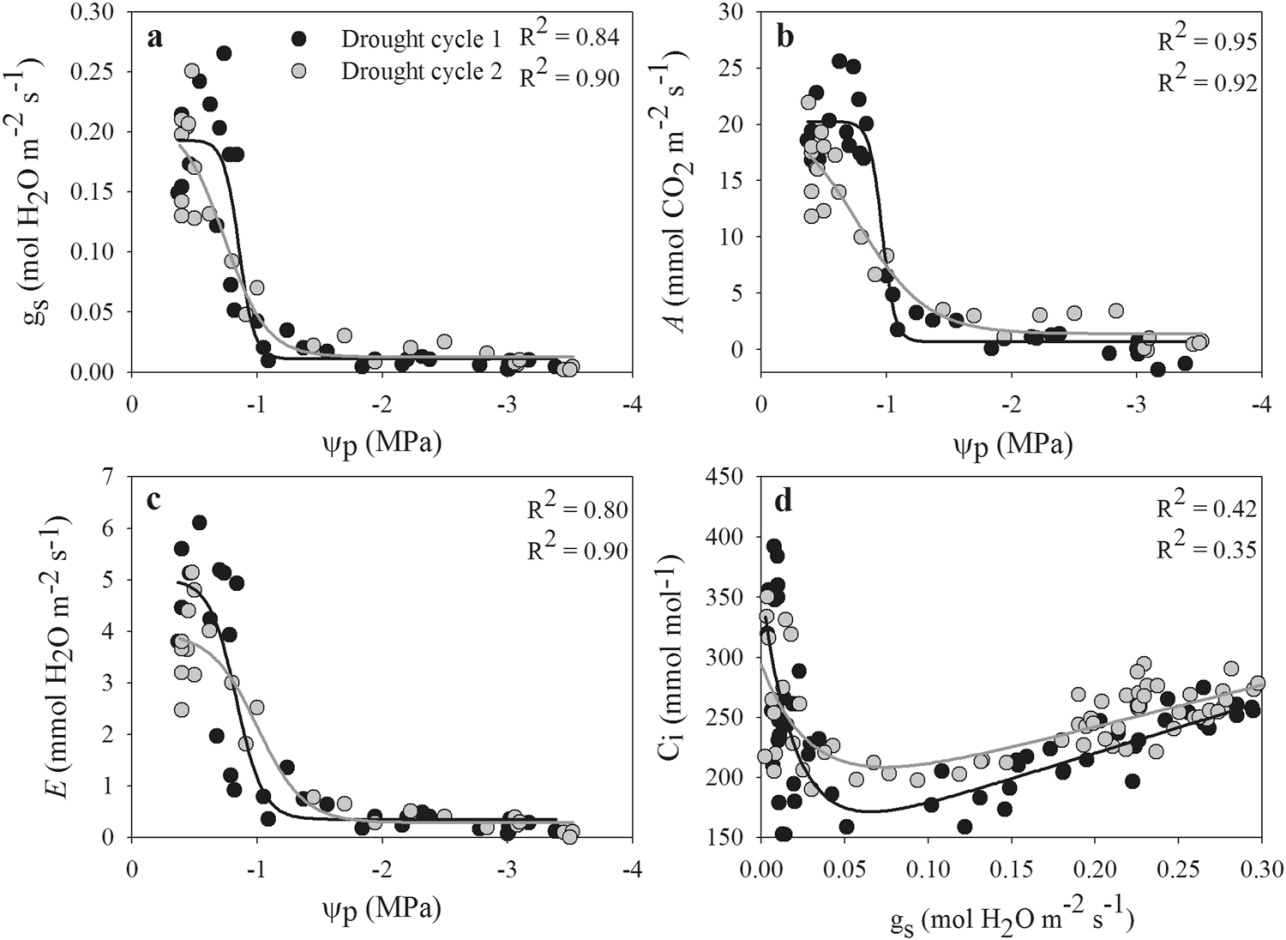
Relationships of measured g_s_, *A* and *E* with Ψ_p_ and Ci with g_s_ in *R. pseudoacacia* of drought plants during drought cycles 1 (black circles) and 2 (grey circles).

A similar pattern of gas exchange response to Ψ_p_ during the drought cycles was observed in both species, which were fit with exponential sigmoid models. Gas exchange declined sharply in a narrow range of Ψ_p_ under drought stress (Figs 2 and 3). The relationship between gas exchange and Ψ_p_ showed some differences between the two drought cycles. In the second drought, *A. fruticose* exhibited a lower intracellular CO_2_ concentration (Ci) when g_s_ < 0.10 mol H_2_O m^−2^ s^−1^ but a higher Ci when g_s_ > 0.10 mol H_2_O m^−2^ s^−1^. In contrast, during the second drought, *R. pseudoacacia* had higher Ci across a broader range of g_s_ values (ca 0.05–0.30 mol H_2_O m^−2^ s^−1^) (Figs 2d and 3d).

**Figure 4:**
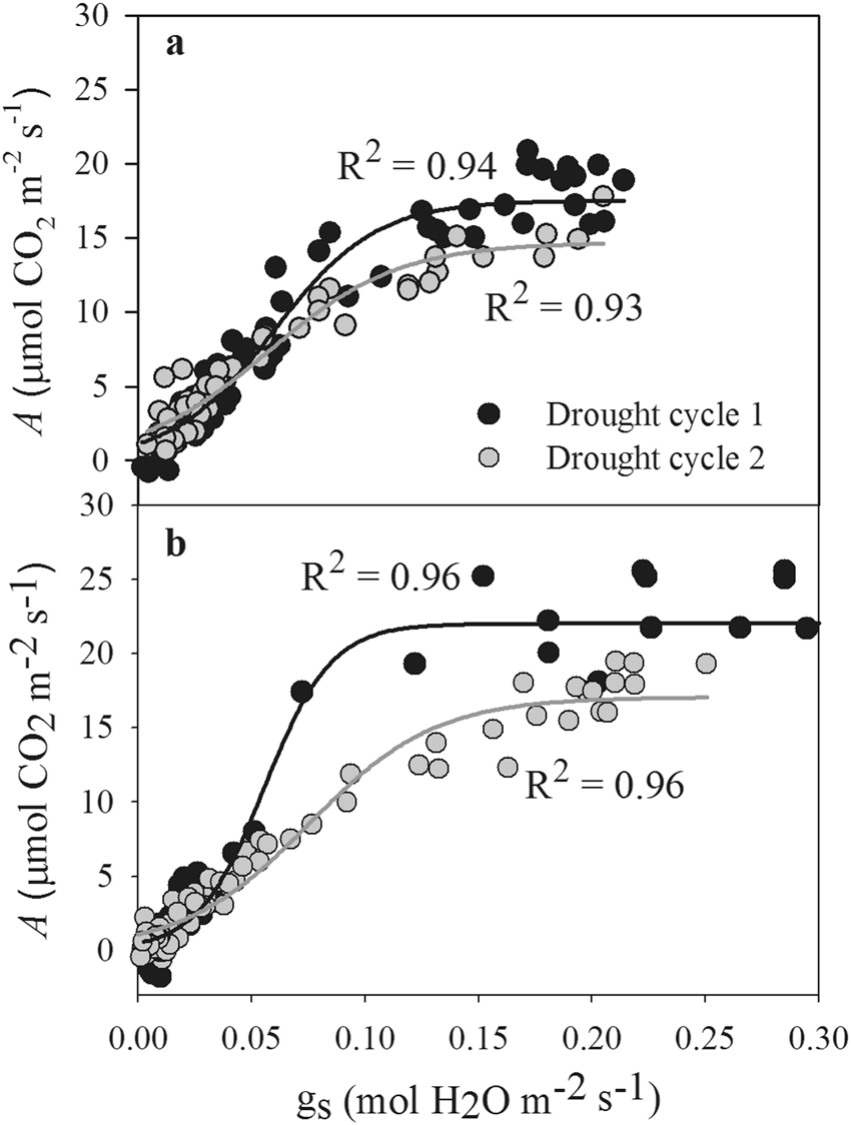
Relationship between *A* and g_s_ during drought cycles 1 (black circles) and 2 (gray circles) in *A. fruticosa* (**a**) and *R. pseudoacacia* (**b**).

The relationship between g_s_ and *A* was well represented by a sigmoidal function in both species and both drought cycles (Fig. 4). The decrease in g_s_ explained more than 90% of the decrease in *A*, indicating a close relationship between *A* and g_s_. Moreover, the relationship between *A* and g_s_ exhibited a shift between the two drought cycles; when g_s_ reached a maximum, the value of *A* was higher in the first drought cycle than in the second drought cycle in both species.

**Figure 5:**
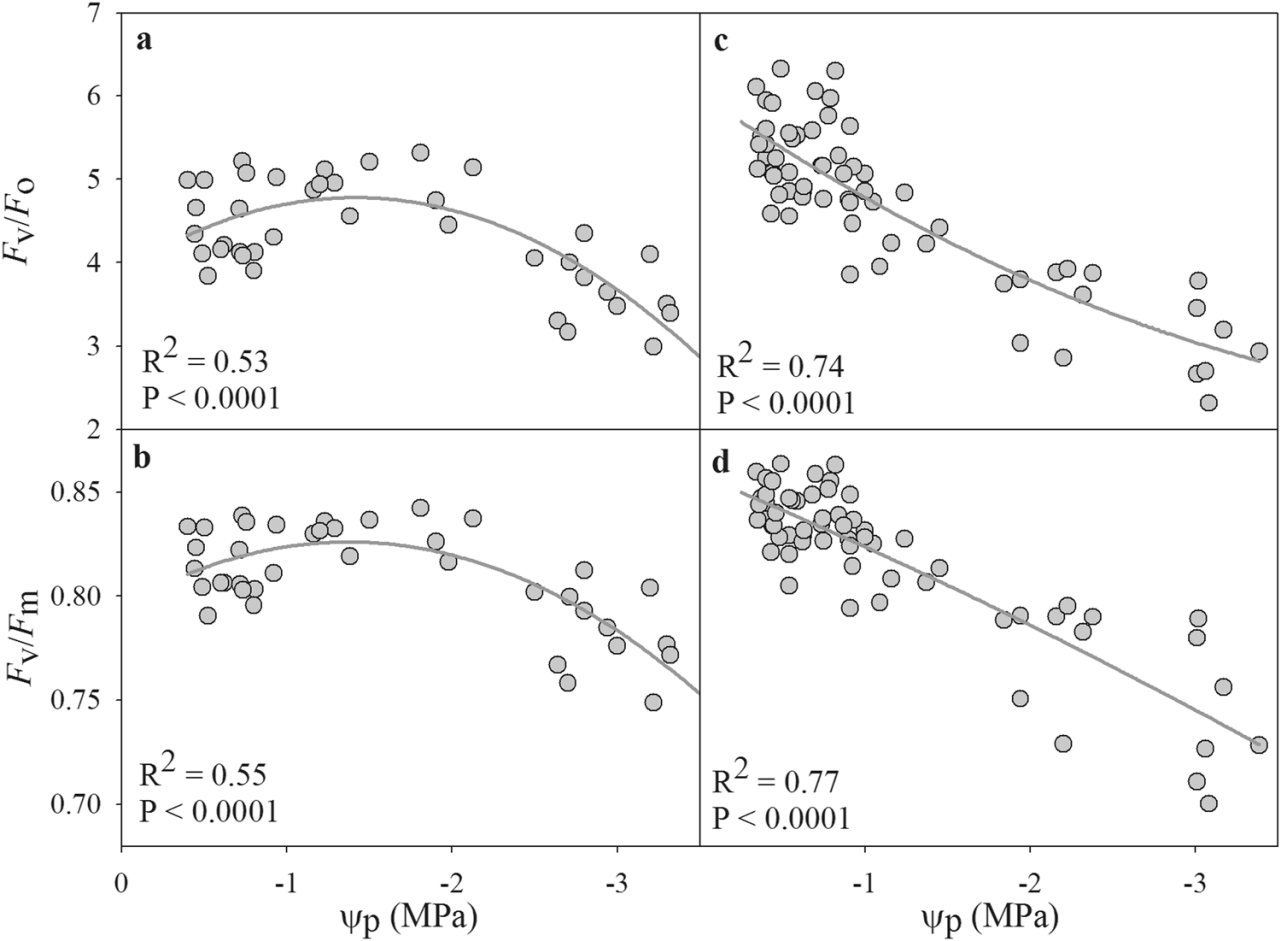
Relationship between the ratio of variable fluorescence to original fluorescence (*F*_v_/*F*_o_) and maximal quantum yield of PS II (*F*_v_/*F*_m_) and the Ψ_p_ during drought in *A. fruticosa* (**a** and **b**) and *R. pseudoacacia* (**c** and **d**). The data used in the figure are pooled over both drought cycles.

Because *F*_v_/*F*_o_ and *F*_v_/*F*_m_ showed no significant difference in the two drought cycles, we pooled the data from the two drought cycles together, and the results indicated a close relationship of *F*_v_/*F*_o_ and *F*_v_/*F*_m_ with Ψ_p_ under drought in both species (Fig. 5). In *A. fruticosa*, the *F*_v_/*F*_o_ and *F*_v_/*F*_m_ ratios first increased and then decreased with drought, and the maximal values occurred at −1.4 MPa. These results indicated that the loss of leaf water in this species did not affect the function of PSII in the early drought stage, in contrast to the decrease in *R. pseudoacacia* with the decline in soil water.

**Figure 6:**
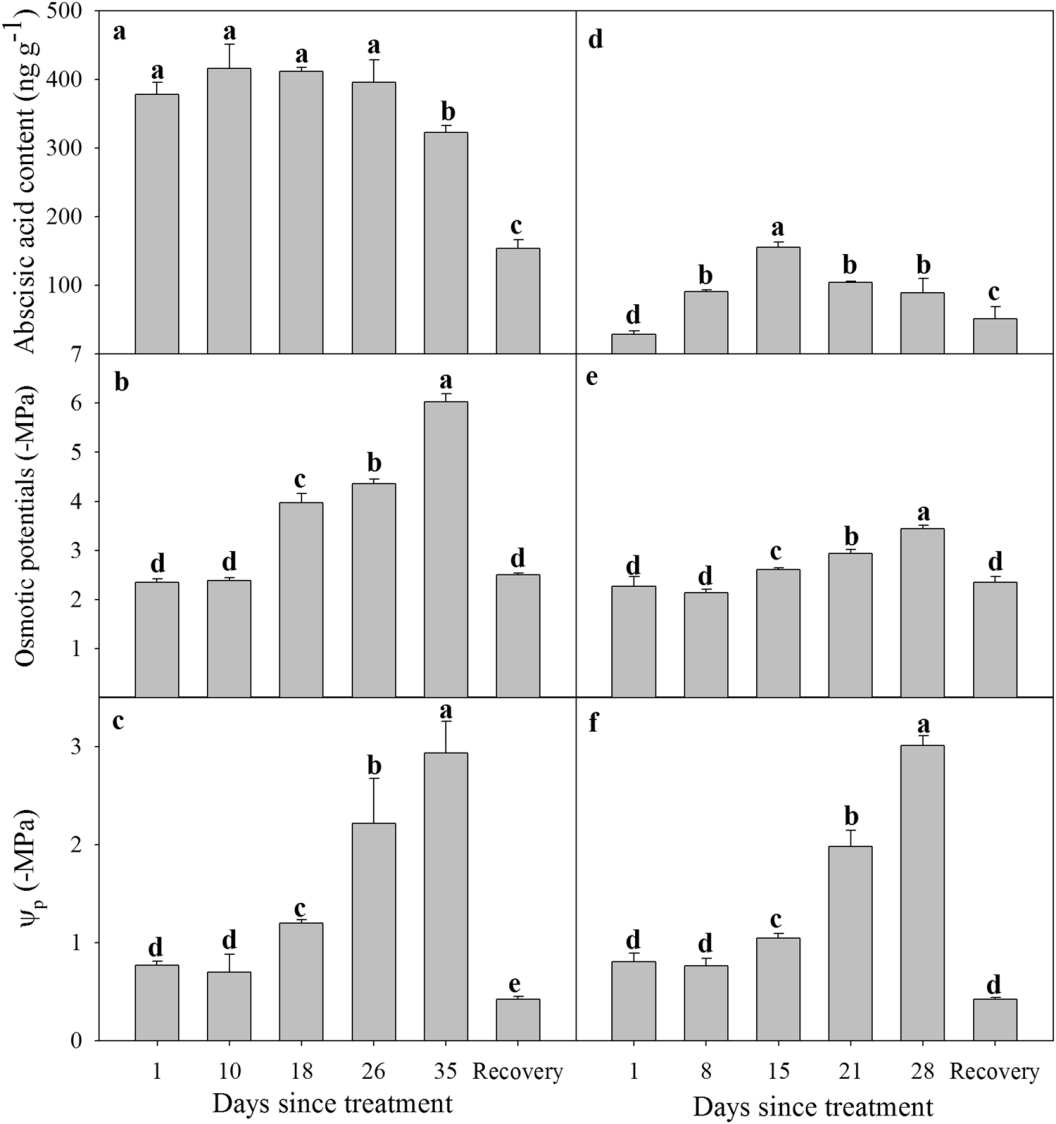
Abscisic acid content, osmotic potential and Ψ_p_ on different days in the first drought cycle and after 7 days of recovery in *A. fruticosa* (**a**,**b**,**c**) and *R. pseudoacacia* (**d**,**e**,**f**). Lowercase letters indicate statistical significance at P < 0.05.

A higher ABA content was observed in *A. fruticosa* than in *R. pseudoacacia* (Fig. 6). In *R. pseudoacacia*, ABA content increased at the beginning of the drought and declined as Ψ_p_ decreased further during the first drought. However, no significant change in ABA content was seen in *A. fruticosa* until severe drought. Both species demonstrated a decrease of ABA when re-watered. Osmotic potential increased with decreasing Ψ_p_ and showed a significant increase in both species. *A. fruticosa* showed a higher osmotic potential than *R. pseudoacacia* under severe drought stress, and the osmotic potential decreased to control levels after re-watering.

### Recovery from drought

**Figure 7:**
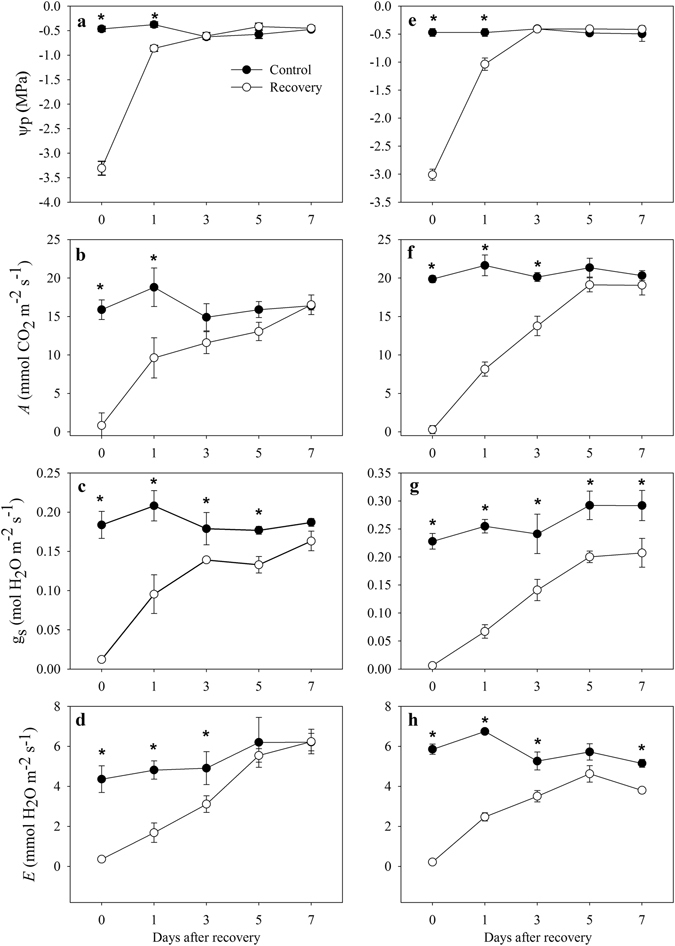
The Ψ_p_, g_s_, *A* and *E* on different days after re-watering following severe drought in *A. fruticosa* (**a**,**b**,**c** and **d**) and *R. pseudoacacia* (**e**,**f**,**g** and **h**). Asterisks indicate statistical significance at P < 0.05 between the control and recovery plants.

Before re-watering, the soil water content was approximately 35% of the field capacity, and in drought-stressed *A. fruticosa* and *R. pseudoacacia* plants, the Ψ_p_ values reached −3.31 and −3.01 MPa, respectively. Moreover, gas exchange parameters were significantly decreased in the saplings subjected to drought relative to those of the control plants before re-watering. After 1 day of recovery, the Ψ_p_ values recovered to −0.86 and −1.06 MPa in *A. fruticosa* and *R. pseudoacacia*, respectively (Fig. 7), and recovered to control levels after 3 days of recovery. The slower recovery of Ψ_p_ in drought-stressed plants affected the recovery of leaf gas exchange in the two species. *A* recovered to control values after 3 and 5 days in both species, but g_s_ did not fully recover after 7 days, and *E* recovered to control levels only in *A. fruticosa*.

**Figure 8:**
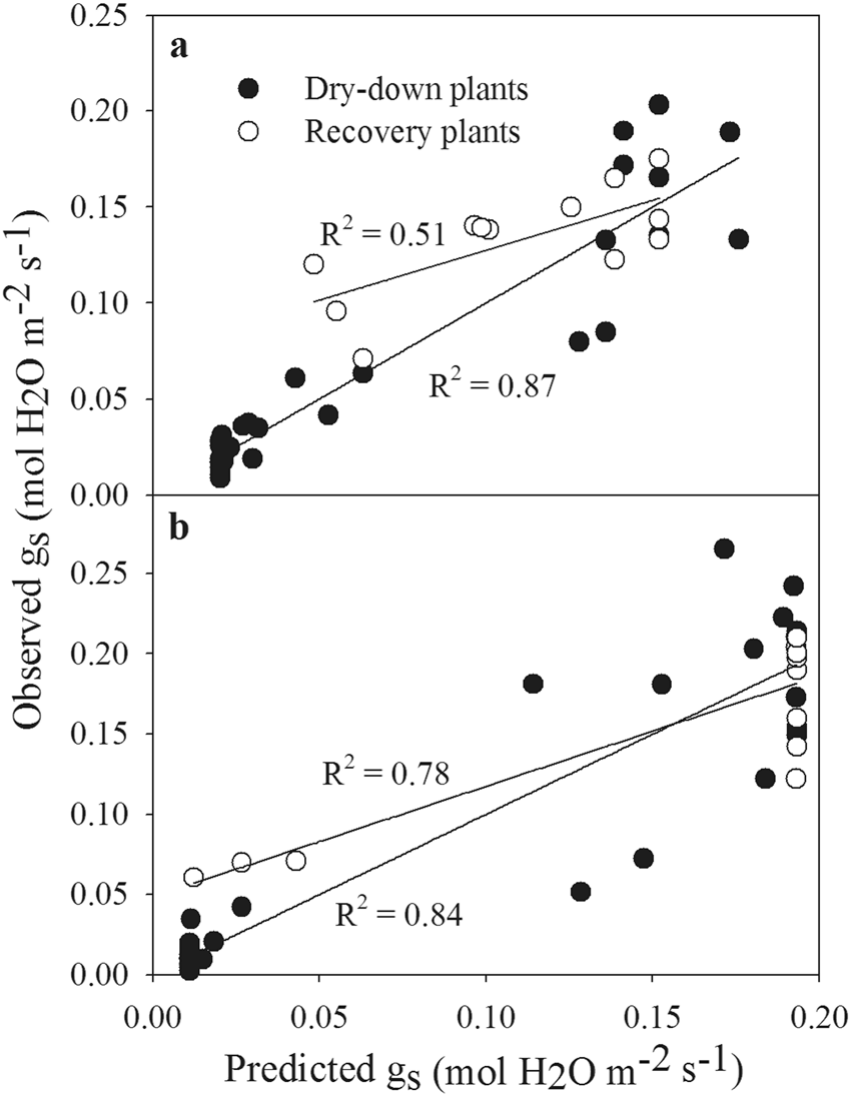
Relationship between predicted and observed g_s_ in plants in the dry-down and recovery groups. There was a significant difference in the slopes between the recovery and dry-down datasets. All of the plants showed a strong correlation (r^2^) between the observed and predicted g_s_ (P < 0.05).

**Figure 9:**
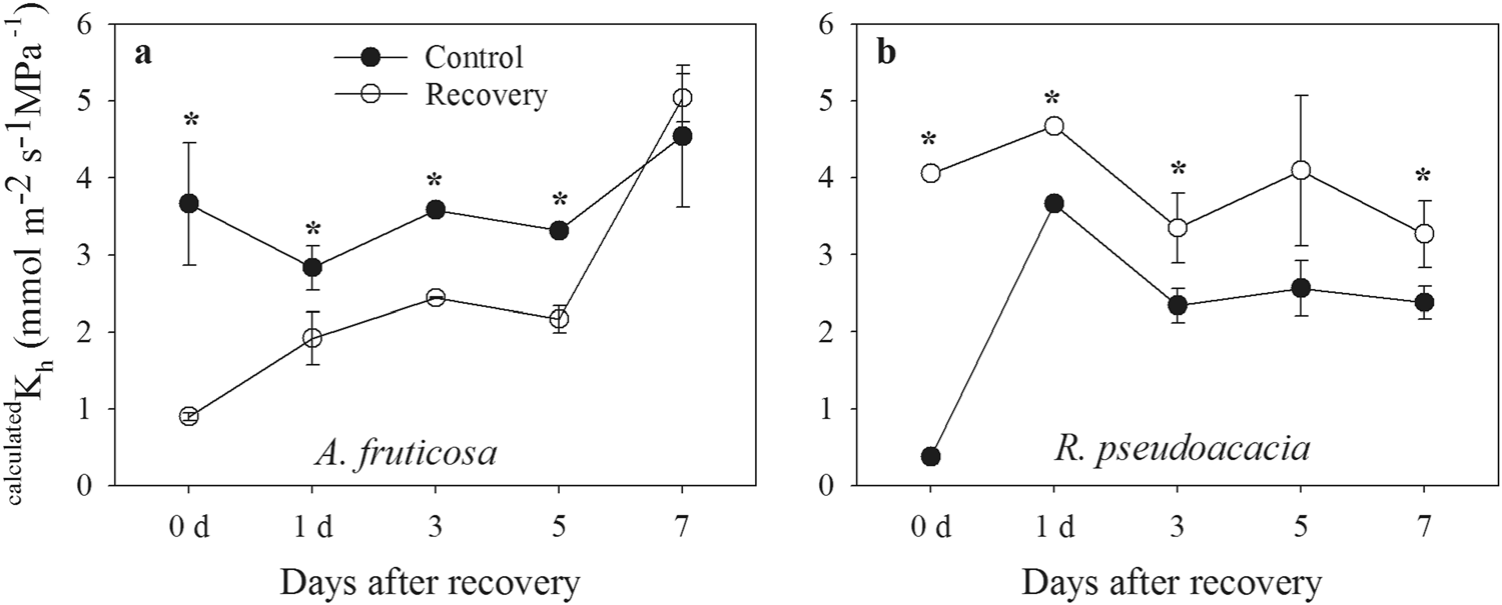
Time-course of whole-plant bulk hydraulic conductance ^calculated^K_h_, calculated according to Ohm’s analog law, K_h_ = *E*/(Ψ_p_ − Ψ_leaf_), measured after re-watering.

A synthesis of the data from all plants in the recovery and dry-down groups revealed a high correspondence between the observed and predicted g_s_ (Fig. 8). The predicted g_s_ was calculated by entering the measured values of Ψ_p_ into the equations obtained from Figs 2a and 3a in the first drought cycle. Regressions of observed g_s_ versus predicted g_s_ showed linear functions and revealed significant differences between the plants in the recovery and dry-down groups (P < 0.01). Bulk hydraulic conductance (K_h_) after 7 days of recovery also showed no difference between the plants in the recovery and control groups in *A. fruticosa* (Fig. 9), suggesting that resistance to water transport is closely aligned with Ψ_leaf_.

## Discussion

### Physiological response of the two species to recurrent drought

The two species investigated in this study are preferred species for afforestation because of their high drought resistance, and have been planted widely in the Loess Plateau region. During both drought cycles, Ψ_p_ declined in both species in parallel with gas exchange due to the course of soil water depletion, which is typical of plants’ responses to drought^28, 30^. In both species, gas exchange parameters were more sensitive than Ψ_p_ to a decrease of the soil water content (Fig. 1). In addition, the results showed that a decrease of g_s_ occurred earlier than the responses of Ψ_p_ and *A* to drought (Fig. 4) because the reduction of g_s_ protects plants against the early loss of Ψ_leaf_ and leaf hydration, which subsequently trigger the further closing of the stomata^16, 31^. Therefore, a significant correlation between g_s_ and Ψ_p_ is expected, as reported in previous studies^16–18^. In the present study, we observed significant correlations between Ψ_p_ and gas exchange in both drought cycles, which indicated that the relationships between Ψ_p_ and gas exchange were not uncoupled across recurrent drought cycles^11, 21^. Upon the decrease of Ψ_p_ to a threshold value, g_s_ sharply declined in parallel with *A* and *E* (Figs 2 and 3). In *R. pseudoacacia* plants, gas exchange showed a slower decline in the second drought cycle and shifted the relationship between Ψ_p_ and gas exchange to the right, and the plants held their stomata open at a lower water potential than in the first drought to maintain higher photosynthesis^7^. In addition, gas exchange and water potential showed no significant changes in the control saplings during the experiment; thus, the shortage of soil water in saplings subjected to drought was responsible for the observed declines in leaf gas exchange and water potential.

Stomata control the exchange of water vapor and CO_2_ between plants and the environment and play an important role in balancing the uptake of CO_2_ for photosynthesis against transpiration loss in plants^15, 32, 33^. In this study, we observed a strong relationship between g_s_ and *A* in both species and during both drought cycles (Fig. 4). In this relationship, *A* reached a plateau at the highest g_s_ values, suggesting that *A* is limited either by the amount of Rubisco or its activity, or by the rate of electron transport^34^. However, with ongoing drought, the decline of g_s_ resulted in proportional decline of *A* in both drought cycles (Fig. 4), which supports the recently proposed conceptual model of photosynthetic limitation under drought^26^. This result indicates that decreased CO_2_ diffusion caused by the decline of g_s_ and mesophyll conductance is responsible for the decline in *A* in early drought, whereas both diffusion and biochemical limitation contributed to the decline of *A* when the soil water content decreased further (Fig. 5), as reported in previous studies^27, 28^. In addition, the value of *A* was higher in the first drought cycle than in the second drought cycle when g_s_ values peaked, indicating that the instantaneous water-use efficiency decreased due to the lower level of *A* when plants experienced drought.

Under drought stress, extensive ABA-mediated stomatal closure occurs, which decreases CO_2_ concentrations^25^. In the present study, we found that the ABA content was significantly higher in *A. fruticosa* than in *R. pseudoacacia*. Our results suggest that, similar to the response of *Cistus albidus* shrubs^35^, ABA did not accumulate at the onset of stress in *A. fruticosa*. In contrast, *R. pseudoacacia* showed a significant increase in ABA content at the onset of drought stress, which indicated a typical stomatal response to drought stress^36^, as the rapid increase in ABA in leaves prevented the opening of stomata when drought intensified (Fig. 6d). However, the ABA-dominated response to drought in *R. pseudoacacia* was not observed in *A. fruticosa* (Fig. 6a), indicating a shift in *A. fruticosa* from ABA-dependent stomatal closure to water potential-dependent stomatal control (Fig. 6) during drought stress^25, 35^ and suggesting the existence of different stomatal closure strategies in the two species. Both species showed a marked decline in ABA with sustained water stress, which indicated that the main driver of stomatal closure was water potential-dependent stomatal control under severe drought (Fig. 6), as suggested by Brodribb and McAdam^25^. In addition, the leaf gas exchange response to Ψ_p_ occurred over a narrow range in both species, indicating that response of gas exchange to drought involved osmotic adjustment and ABA signaling^25^.

### Dynamic recovery from drought in both species

After re-watering, Ψ_p_ recovered to control levels more rapidly than the gas exchange parameters (Fig. 7), consistent with previous reports^29, 31^. However, compared with Ψ_p_, some hysteresis of leaf gas exchange recovery was observed after re-watering (Fig. 7), and g_s_ presented 90% and 70% recovery in *A. fruticosa* and *R. pseudoacacia*, respectively. These findings are consistent with previous work indicating a partial recovery of g_s_^29, 37^ and suggest that the regulation of leaf gas exchange might also be mediated by the residual ABA signal during the recovery stage (Fig. 6), especially in *R. pseudoacacia*, and that the recovery of water potential was potentially promoted by limiting the opening of the stomata after re-watering, possibly due to the transport of ABA accumulated in the roots during drought to the leaves after re-watering, along with reduced transpiration via stomatal control^29^. However, *A* in stressed plants after 5 days of recovery showed no significant difference from control levels, which indicated that plants were able to maintain a value of *A* similar to that of the control plants after re-watering (Fig. 7).

**Figure 10:**
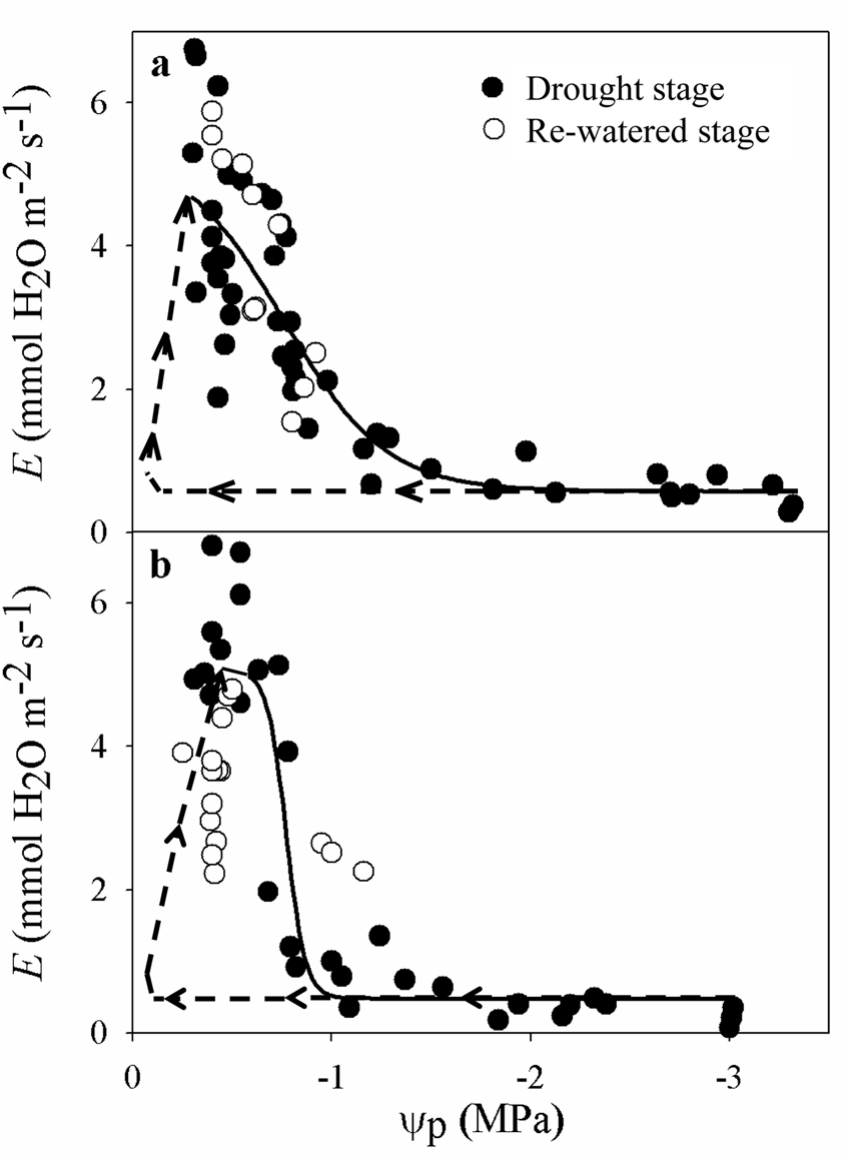
Relationship between *E* and the Ψ_p_ in drought-stressed plants (black circles) and re-watered plants (white circles) after re-watering in *A. fruticosa* (**a**) and *R. pseudoacacia* (**b**). The solid line represents the decrease in *E* as Ψ_p_ decreased, and the dashed line represents the theoretical non-hydraulic limited recovery following the model proposed by Brodribb and Cochard^7^.

Previous studies have indicated that hydraulic limitations control the recovery of leaf gas exchange in plants after re-watering following drought stress^29, 38^. The assessment of xylem failure in branches requires large portions of branches, which can affect the hydraulic functioning of the plants; however, the absence of hydraulic failure can be inferred from the recovery of *E* under drought treatment^7, 29, 38^. In the present study, we obtained evidence that hydraulic limitation governed the recovery of gas exchange following drought stress in both species. The K_h_ values of plants in the recovery group reached levels similar to those of control plants after 5 days of recovery (Fig. 9), showing that the hydraulic functionality of plant organs recovers rather slowly compared with the Ψ_p_, which is not sufficient to restore leaf transpiration. In the present study, a hydraulic-stomatal limitation model was confirmed in both woody angiosperm species under recovery from severe drought (Fig. 10), which has also been demonstrated in previous studies^7^. According to the function describing the relationship between Ψ_p_ and *E*, both during drought and post-drought (Fig. 10), changes in ABA appeared to play no role in stomatal recovery from drought in either species in the re-watered stage. This finding was consistent with the results of Wilkinson and Davies^39^ and Brodribb and Cochard^7^, as Ψ_p_ rapidly increased to zero after re-watering under ABA-induced non-hydraulic limitation, due to a lower *E* and saturated soil, which was not observed in either of the two species examined in the present study, suggesting that the stomatal recovery of both species is mediated by factors affecting the transport of water from the soil to the points of photosynthesis. In this study, we did not observe hysteresis between the plants in the drought and recovery groups in the *E* versus Ψ_p_ curve in the woody shrub *A. fruticosa*, indicating that the recovery of *E* in *A. fruticosa* was constrained by the hydraulic system, as suggested by Brodribb and Cochard^7^. However, in the tree species *R. pseudoacacia*, we observed hysteresis of the *E* versus Ψ_p_ curve in the later recovery stage, which indicated that hydraulic and non-hydraulic limitations may be involved in the recovery of gas exchange (Figs 6, 8, 9 and 10)^7, 29^. This result differs from the results obtained in conifers, as the recovery of conifers from drought stress conforms to a hydraulic limitation pattern and shows no hysteresis^7^, but is consistent with the limitation pattern observed in *Eucalyptus pauciflora* after re-watering, which shows hysteresis^29^. The limitations of the recovery of gas exchange in both species warrant further study.

In conclusion, drought stress reduced the leaf water potential and gas exchange in both species, with gas exchange being more sensitive to drought than the water potential. Additionally, a strong relationship was observed between the Ψ_p_ and gas exchange during both drought cycles, which showed a shift to the right in the second drought cycle in *R. pseudoacacia*. Furthermore, stomatal closure was mainly regulated by ABA content in *R. pseudoacacia* in early drought stages, but not in *A. fruticosa*, which showed a shift from ABA-driven to water-potential-driven stomatal closure. After re-watering, the Ψ_p_ recovered more rapidly than did gas exchange. The recovery pattern of transpiration in *A. fruticosa* and *R. pseudoacacia* suggested that the recovery of gas exchange from severe drought is mainly restricted by hydraulic factors in both species; however, non-hydraulic factors may also be involved in *R. pseudoacacia*. Our results revealed a shift in the close relationship between Ψ_p_ and gas exchange in consecutive drought cycles and the recovery mechanism following severe drought in *A. fruticosa* and *R. pseudoacacia*.

## Materials and Methods

### Plant cultivation and growth conditions

This study was undertaken in an open-sided rain-shelter with a glass roof at the Institute of Soil and Water Conservation in Yangling, Shaanxi Province (34°17′N, 108°04′E). The experimental region has a temperate and sub-humid climate, with a mean annual temperature of 13 °C and mean annual precipitation of 632 mm, of which approximately 60% occurs in July–September.

Two-year old seedlings were used of two deciduous, woody legume species: *A. fruticosa* L. (shrub) and *R. pseudoacacia* L. (tree). Seeds of both plants had been sown at the same time in the nursery in the previous year. Three months before the initiation of the experiment, *A. fruticosa* (30–50 cm tall and 3–5 mm in diameter at the stem base) and *R. pseudoacacia* (40–60 cm tall and 3–5 mm in diameter at the stem base) were transplanted from the field to 500-L pots (980 × 760 × 680 mm, length × width × height), which could improve the reliability of the experimental results because small plots may change the experimental results and compromise the experiment^40^. Soil was collected from the 0–20-cm soil layer.

A total of 24 plants of each species were assessed during two drought cycles and were divided into two groups using a completely randomized design: well-watered saplings (control treatment) and non-watered saplings (drought treatment), with three replications. The control and drought saplings were each subjected to both drought periods. All of the plants were well watered until the start of the experiment. The control saplings continued to be well watered throughout the experimental period. Drought stress was induced by ceasing watering until the leaf photosynthesis rate declined to zero or the Ψ_p_ decreased to between −3.0 and −3.5 MPa, which required approximately five and four weeks in *A. fruticosa* and *R. pseudoacacia*, respectively. The plants were then re-watered to approximately field capacity until net photosynthesis had almost completely recovered, i.e., after approximately one week. The stressed saplings were maintained without watering for the second drought cycle. Throughout the experiment, the Ψ_leaf_, gas exchange and chlorophyll fluorescence were measured in leaves exposed to sun on the upper crown of the plants. Measurements were obtained from at least three plants in each replicate. Soil water content (SWC) was observed with SWC reflectometers (CS650-L, Campbell Scientific, Australia). Soil moisture at 10 and 40 cm was recorded every 30 minutes, and the average SWC was calculated.

### Determination of gas exchange and chlorophyll fluorescence

Gas exchange parameters were measured in mature, fully expanded leaves from the upper crown of plants. Gas exchange and chlorophyll fluorescence were measured in the same leaf. Gas exchange traits, including the net photosynthesis rate (*A*), transpiration rate (*E*), stomatal conductance (g_s_) and intracellular CO_2_ concentration (Ci), were recorded by a Li-Cor model 6400 system (Lincoln, NE, USA). At least three plants per replicate (two leaves per plant) were selected during 09:00–11:00 h. The saturating photosynthetic photon flux density was between 1000 and 1500 μmol m^−2^ s^−1^ in the leaf chamber during the measurement periods, and the temperature, CO_2_ concentration and relative humidity inside the leaf cuvette were always close to ambient air values.

A portable pulse-amplitude-modulated fluorometer was used to measure the chlorophyll fluorescence parameters with an FMS-2.02 system (Hansatech Instruments, Norfolk, UK). The initial and maximum fluorescence (*F*_o_ and *F*_m_) were recorded after 30 minutes of darkness, and the following parameters were determined and calculated: *F*_v_/*F*_m_, the maximum quantum efficiency of photosystem II, which was used to assess the potential maximal quantum yield; and *F*_v_/*F*_o_, the ratio of variable fluorescence to original fluorescence, which represented the PS II activity.

### Water relations

Spot Ψ_p_ was determined in both control and stressed plants between 05:00 and 06:00 h using a PMS 600 pressure chamber (PMS Instruments Company, Albany, USA). After the measurement of gas exchange, Ψ_leaf_ was measured, and leaf samples were collected, wrapped in aluminum foil, immediately frozen in liquid N_2_ for ten minutes, then transferred to a −80 °C freezer for additional analyses.

### Determination of leaf abscisic acid concentrations and osmotic potentials

ABA was extracted according to the method reported by Luo *et al*.^41^. First, the fresh samples were finely ground with liquid N_2_ and then extracted using 4 mL of an ice-cold solution containing 80% methanol, 500 mg L^−1^ citric acid monohydrate and 200 mg L^−1^ butylated hydroxytoluene. Each sample was shaken overnight at 4 °C and then centrifuged for 15 minutes at 4 °C and 10,000 g. The supernatant was collected. We repeated the above process twice, pooled the supernatants and dried them using N_2_ gas. The residual dried compounds were dissolved using 800 µL 80% methanol and then filtered using a 0.22-µm organic membrane. Extracted samples were analyzed using a high-performance liquid chromatography unit (LC-20AT, Shimadzu, Kinh Do, Japan). ABA standards (A1049) were used for the quantification of hormone concentrations and were purchased from Sigma (St Louis, MO, USA). The leaf osmotic potential was measured using a Model 5600 dew point microvolt meter (Logan, UT, USA).

### Statistical analyses

The response of stomata to Ψ_leaf_ was a main component of the hydraulic model. The relationship between gas exchange and Ψ_p_ was determined using SigmaPlot software (SPSS Inc., Chicago, IL, USA) to fit a 4-parameter sigmoid function of the form y = a + b/(1 + e^(−(Ψp −c)/d)^) to the pooled gas exchange parameters versus Ψ_p_ data collected from each plant during recurrent drought, where a, b, c and d are fitted parameters of the 4-parameter sigmoid function, and y represents the gas exchange parameters. The predicted g_s_ was calculated by entering the measured values of Ψ_p_ from the first drought stage and the re-watered stage into the regression equations, which were established using pooled g_s_ and Ψ_p_ data from the first drought cycle for both species. Moreover, Ψ_p_ was employed to estimate the plant bulk hydraulic conductance (K_h_), calculated according to Ohm’s analog law, K_h_ = *E*/(Ψ_p_ − Ψ_leaf_)^42^, and Ψ_p_ was taken as a proxy for Ψ_soil_. Independent-samples t tests were used to test the statistical significance of the differences in the dynamics of physiological parameters under recurrent drought between the plants in the recovery and control groups during the re-watered stage. A general linear model was employed to compare the differences in observed and predicted g_s_ between the plants in the recovery and dry-down groups (SPSS Inc., Chicago, IL, USA).
